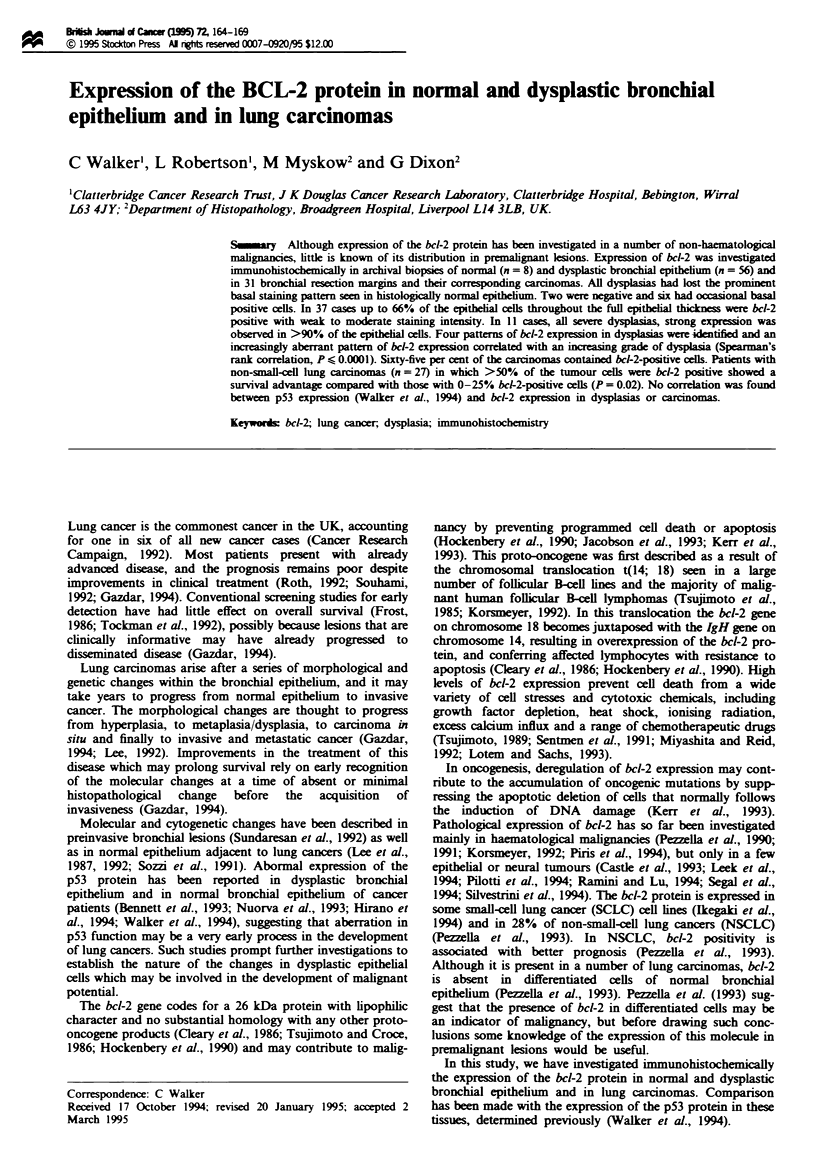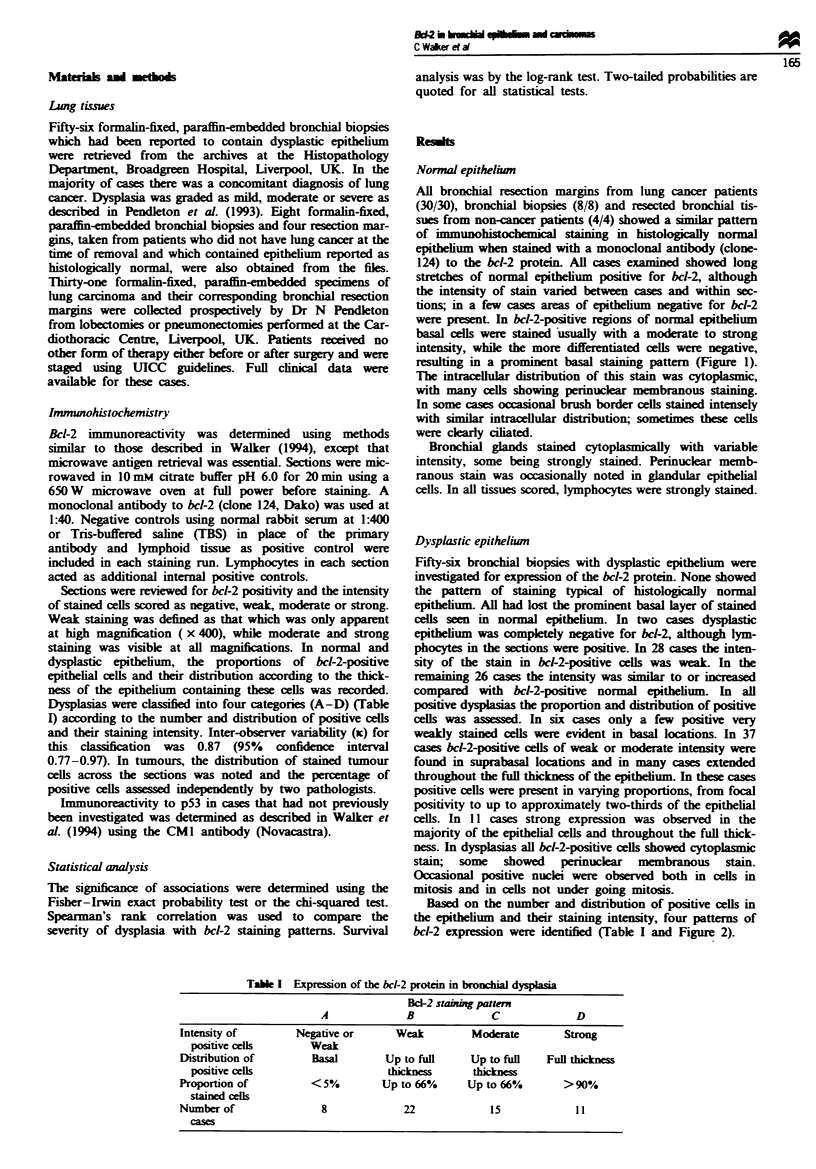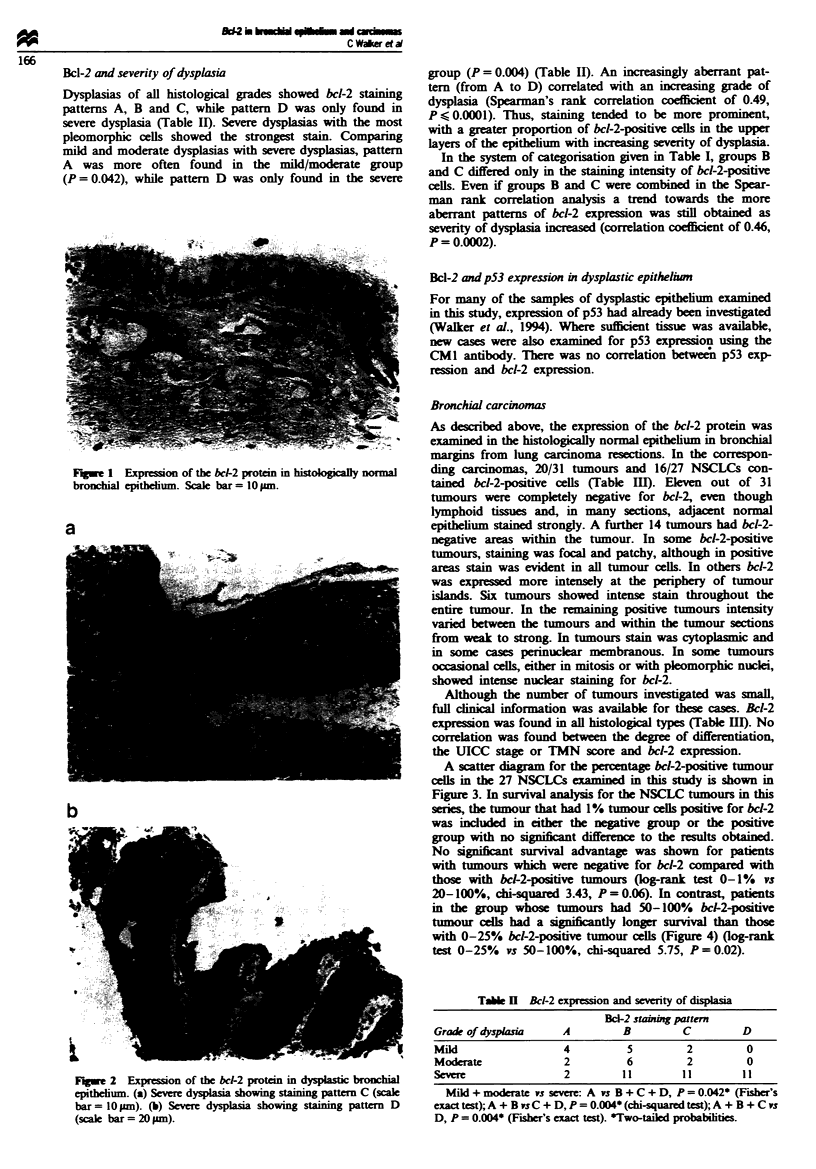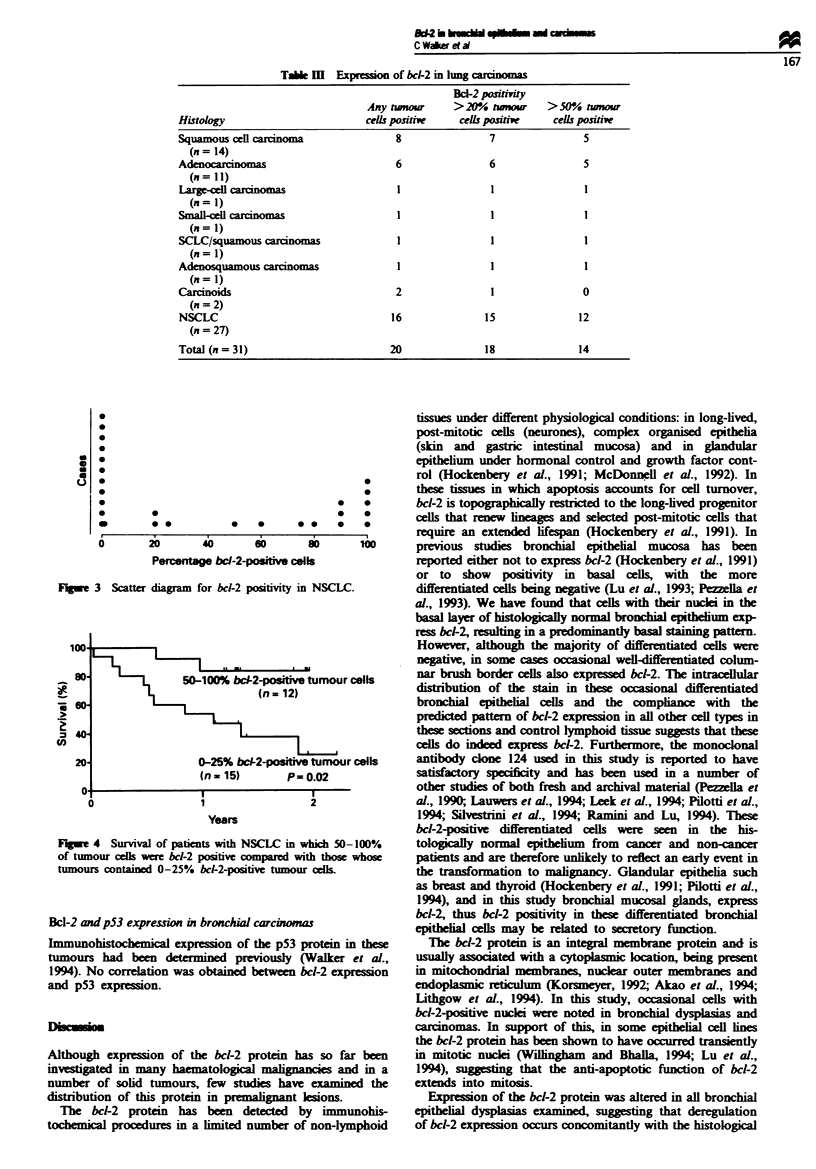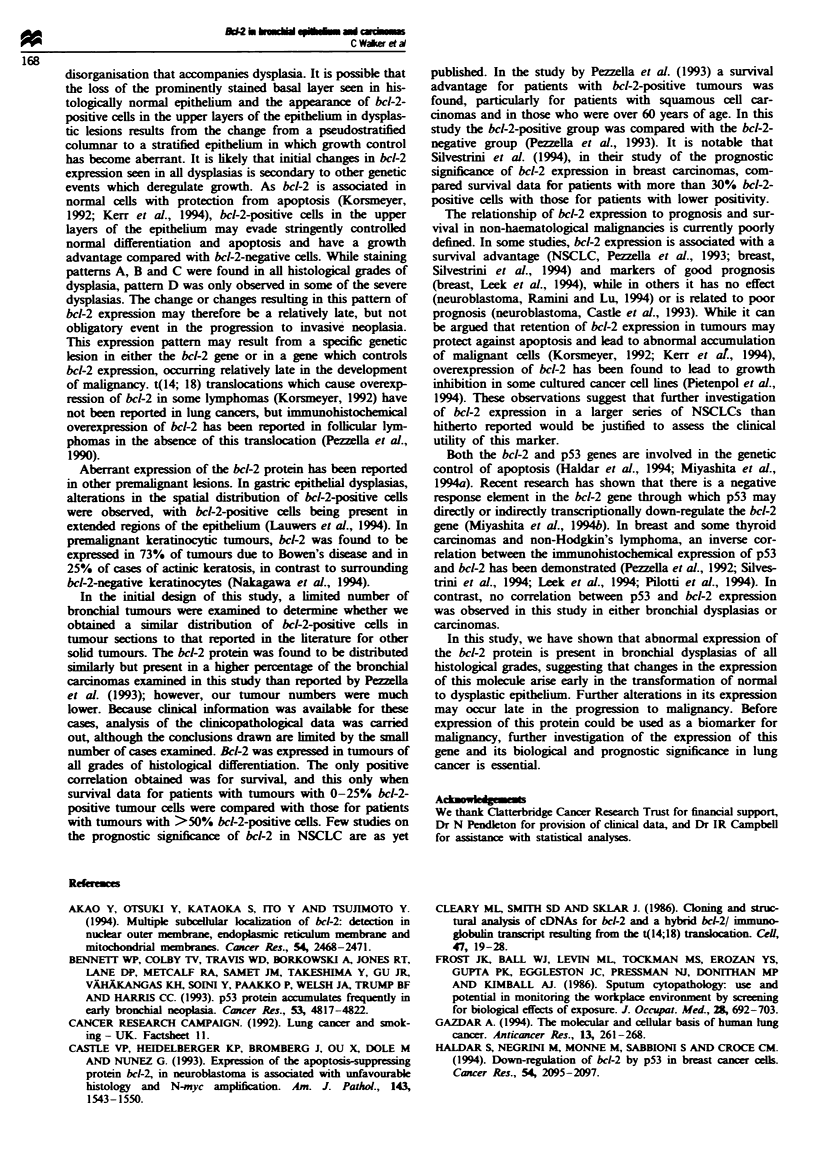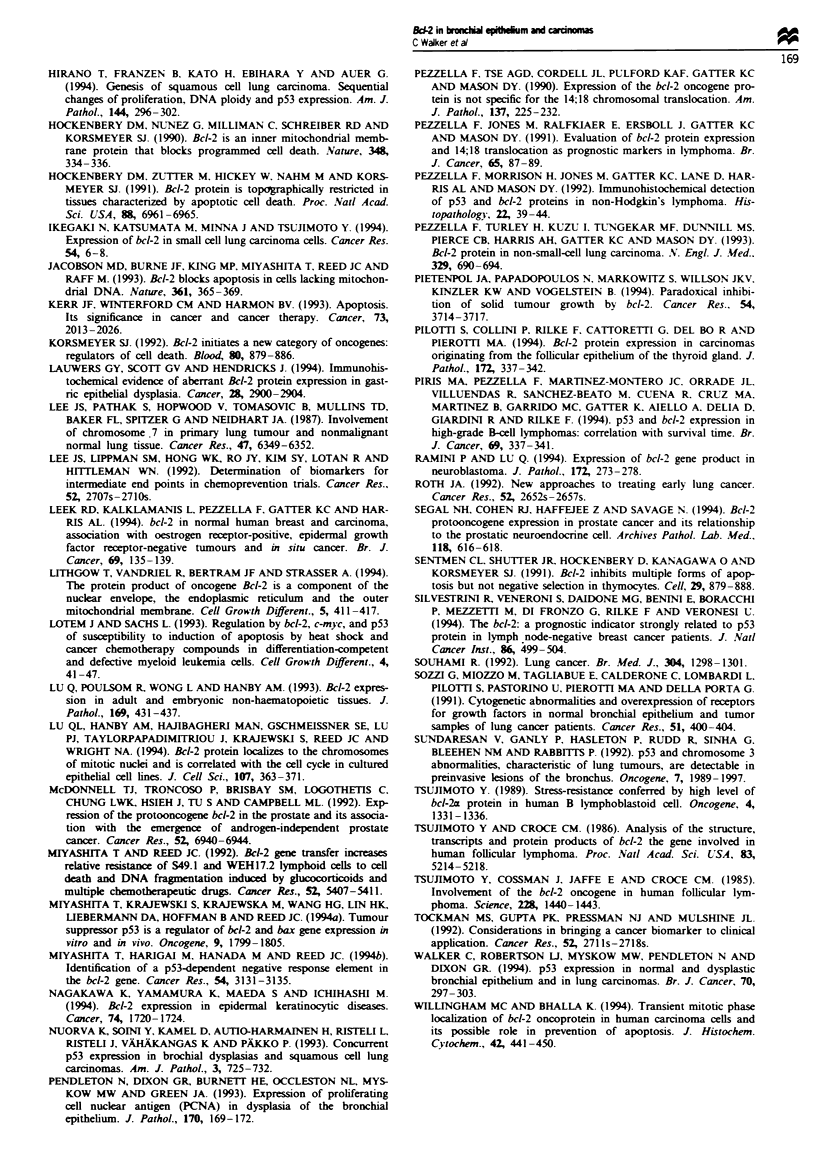# Expression of the BCL-2 protein in normal and dysplastic bronchial epithelium and in lung carcinomas.

**DOI:** 10.1038/bjc.1995.295

**Published:** 1995-07

**Authors:** C. Walker, L. Robertson, M. Myskow, G. Dixon

**Affiliations:** Clatterbridge Cancer Research Trust, J K Douglas Cancer Research Laboratory, Clatterbridge Hospital, Bebington, Wirral, UK.

## Abstract

**Images:**


© 1995 Stockton Press All rights reserved 0007-0920/95 $12.00

### Expression of the BCL-2 protein in normal and dysplastic bronchial epithelium and in lung carcinomas

C Walker[1], L Robertson[1], M Myskow[2] and G Dixon[2]

[1]*Clatterbridge Cancer Research Trust, J K Douglas Cancer Research Laboratory, Clatterbridge Hospital, Bebington, Wirral L63 4JY;* [2]*Department of Histopathology, Broadgreen Hospital, Liverpool L14 3LB, UK.*

**Summary** Although expression of the *bcl*-2 protein has been investigated in a number of non-haematological malignancies, little is known of its distribution in premalignant lesions. Expression of *bcl*-2 was investigated immunohistochemically in archival biopsies of normal ($n = 8$) and dysplastic bronchial epithelium ($n = 56$) and in 31 bronchial resection margins and their corresponding carcinomas. All dysplasias had lost the prominent basal staining pattern seen in histologically normal epithelium. Two were negative and six had occasional basal positive cells. In 37 cases up to 66% of the epithelial cells throughout the full epithelial thickness were *bcl*-2 positive with weak to moderate staining intensity. In 11 cases, all severe dysplasias, strong expression was observed in >90% of the epithelial cells. Four patterns of *bcl*-2 expression in dysplasias were identified and an increasingly aberrant pattern of *bcl*-2 expression correlated with an increasing grade of dysplasia (Spearman's rank correlation, $P \leqslant 0.0001$). Sixty-five per cent of the carcinomas contained *bcl*-2-positive cells. Patients with non-small-cell lung carcinomas ($n = 27$) in which >50% of the tumour cells were *bcl*-2 positive showed a survival advantage compared with those with 0-25% *bcl*-2-positive cells ($P = 0.02$). No correlation was found between p53 expression (Walker *et al.*, 1994) and *bcl*-2 expression in dysplasias or carcinomas.

**Keywords:** *bcl*-2; lung cancer; dysplasia; immunohistochemistry

Lung cancer is the commonest cancer in the UK, accounting for one in six of all new cancer cases (Cancer Research Campaign, 1992). Most patients present with already advanced disease, and the prognosis remains poor despite improvements in clinical treatment (Roth, 1992; Souhami, 1992; Gazdar, 1994). Conventional screening studies for early detection have had little effect on overall survival (Frost, 1986; Tockman *et al.*, 1992), possibly because lesions that are clinically informative may have already progressed to disseminated disease (Gazdar, 1994).

Lung carcinomas arise after a series of morphological and genetic changes within the bronchial epithelium, and it may take years to progress from normal epithelium to invasive cancer. The morphological changes are thought to progress from hyperplasia, to metaplasia/dysplasia, to carcinoma *in situ* and finally to invasive and metastatic cancer (Gazdar, 1994; Lee, 1992). Improvements in the treatment of this disease which may prolong survival rely on early recognition of the molecular changes at a time of absent or minimal histopathological change before the acquisition of invasiveness (Gazdar, 1994).

Molecular and cytogenetic changes have been described in preinvasive bronchial lesions (Sundaresan *et al.*, 1992) as well as in normal epithelium adjacent to lung cancers (Lee *et al.*, 1987, 1992; Sozzi *et al.*, 1991). Abormal expression of the p53 protein has been reported in dysplastic bronchial epithelium and in normal bronchial epithelium of cancer patients (Bennett *et al.*, 1993; Nuorva *et al.*, 1993; Hirano *et al.*, 1994; Walker *et al.*, 1994), suggesting that aberration in p53 function may be a very early process in the development of lung cancers. Such studies prompt further investigations to establish the nature of the changes in dysplastic epithelial cells which may be involved in the development of malignant potential.

The *bcl*-2 gene codes for a 26 kDa protein with lipophilic character and no substantial homology with any other proto-oncogene products (Cleary *et al.*, 1986; Tsujimoto and Croce, 1986; Hockenbery *et al.*, 1990) and may contribute to malignancy by preventing programmed cell death or apoptosis (Hockenbery *et al.*, 1990; Jacobson *et al.*, 1993; Kerr *et al.*, 1993). This proto-oncogene was first described as a result of the chromosomal translocation t(14; 18) seen in a large number of follicular B-cell lines and the majority of malignant human follicular B-cell lymphomas (Tsujimoto *et al.*, 1985; Korsmeyer, 1992). In this translocation the *bcl*-2 gene on chromosome 18 becomes juxtaposed with the *IgH* gene on chromosome 14, resulting in overexpression of the *bcl*-2 protein, and conferring affected lymphocytes with resistance to apoptosis (Cleary *et al.*, 1986; Hockenbery *et al.*, 1990). High levels of *bcl*-2 expression prevent cell death from a wide variety of cell stresses and cytotoxic chemicals, including growth factor depletion, heat shock, ionising radiation, excess calcium influx and a range of chemotherapeutic drugs (Tsujimoto, 1989; Sentmen *et al.*, 1991; Miyashita and Reid, 1992; Lotem and Sachs, 1993).

In oncogenesis, deregulation of *bcl*-2 expression may contribute to the accumulation of oncogenic mutations by suppressing the apoptotic deletion of cells that normally follows the induction of DNA damage (Kerr *et al.*, 1993). Pathological expression of *bcl*-2 has so far been investigated mainly in haematological malignancies (Pezzella *et al.*, 1990; 1991; Korsmeyer, 1992; Piris *et al.*, 1994), but only in a few epithelial or neural tumours (Castle *et al.*, 1993; Leek *et al.*, 1994; Pilotti *et al.*, 1994; Ramini and Lu, 1994; Segal *et al.*, 1994; Silvestrini *et al.*, 1994). The *bcl*-2 protein is expressed in some small-cell lung cancer (SCLC) cell lines (Ikegaki *et al.*, 1994) and in 28% of non-small-cell lung cancers (NSCLC) (Pezzella *et al.*, 1993). In NSCLC, *bcl*-2 positivity is associated with better prognosis (Pezzella *et al.*, 1993). Although it is present in a number of lung carcinomas, *bcl*-2 is absent in differentiated cells of normal bronchial epithelium (Pezzella *et al.*, 1993). Pezzella *et al.* (1993) suggest that the presence of *bcl*-2 in differentiated cells may be an indicator of malignancy, but before drawing such conclusions some knowledge of the expression of this molecule in premalignant lesions would be useful.

In this study, we have investigated immunohistochemically the expression of the *bcl*-2 protein in normal and dysplastic bronchial epithelium and in lung carcinomas. Comparison has been made with the expression of the p53 protein in these tissues, determined previously (Walker *et al.*, 1994).

Correspondence: C Walker
Received 17 October 1994; revised 20 January 1995; accepted 2 March 1995

### Materials and methods

### *Lung tissues*

Fifty-six formalin-fixed, paraffin-embedded bronchial biopsies which had been reported to contain dysplastic epithelium were retrieved from the archives at the Histopathology Department, Broadgreen Hospital, Liverpool, UK. In the majority of cases there was a concomitant diagnosis of lung cancer. Dysplasia was graded as mild, moderate or severe as described in Pendleton *et al.* (1993). Eight formalin-fixed, paraffin-embedded bronchial biopsies and four resection margins, taken from patients who did not have lung cancer at the time of removal and which contained epithelium reported as histologically normal, were also obtained from the files. Thirty-one formalin-fixed, paraffin-embedded specimens of lung carcinoma and their corresponding bronchial resection margins were collected prospectively by Dr N Pendleton from lobectomies or pneumonectomies performed at the Cardiothoracic Centre, Liverpool, UK. Patients received no other form of therapy either before or after surgery and were staged using UICC guidelines. Full clinical data were available for these cases.

### *Immunohistochemistry*

*Bcl*-2 immunoreactivity was determined using methods similar to those described in Walker (1994), except that microwave antigen retrieval was essential. Sections were microwaved in 10 mM citrate buffer pH 6.0 for 20 min using a 650 W microwave oven at full power before staining. A monoclonal antibody to *bcl*-2 (clone 124, Dako) was used at 1:40. Negative controls using normal rabbit serum at 1:400 or Tris-buffered saline (TBS) in place of the primary antibody and lymphoid tissue as positive control were included in each staining run. Lymphocytes in each section acted as additional internal positive controls.

Sections were reviewed for *bcl*-2 positivity and the intensity of stained cells scored as negative, weak, moderate or strong. Weak staining was defined as that which was only apparent at high magnification ( × 400), while moderate and strong staining was visible at all magnifications. In normal and dysplastic epithelium, the proportions of *bcl*-2-positive epithelial cells and their distribution according to the thickness of the epithelium containing these cells was recorded. Dysplasias were classified into four categories (A–D) (Table I) according to the number and distribution of positive cells and their staining intensity. Inter-observer variability ($\kappa$) for this classification was 0.87 (95% confidence interval 0.77–0.97). In tumours, the distribution of stained tumour cells across the sections was noted and the percentage of positive cells assessed independently by two pathologists.

Immunoreactivity to p53 in cases that had not previously been investigated was determined as described in Walker *et al.* (1994) using the CM1 antibody (Novacastra).

### *Statistical analysis*

The significance of associations were determined using the Fisher–Irwin exact probability test or the chi-squared test. Spearman's rank correlation was used to compare the severity of dysplasia with *bcl*-2 staining patterns. Survival analysis was by the log-rank test. Two-tailed probabilities are quoted for all statistical tests.

### Results

### *Normal epithelium*

All bronchial resection margins from lung cancer patients (30/30), bronchial biopsies (8/8) and resected bronchial tissues from non-cancer patients (4/4) showed a similar pattern of immunohistochemical staining in histologically normal epithelium when stained with a monoclonal antibody (clone-124) to the *bcl*-2 protein. All cases examined showed long stretches of normal epithelium positive for *bcl*-2, although the intensity of stain varied between cases and within sections; in a few cases areas of epithelium negative for *bcl*-2 were present. In *bcl*-2-positive regions of normal epithelium basal cells were stained usually with a moderate to strong intensity, while the more differentiated cells were negative, resulting in a prominent basal staining pattern (Figure 1). The intracellular distribution of this stain was cytoplasmic, with many cells showing perinuclear membranous staining. In some cases occasional brush border cells stained intensely with similar intracellular distribution; sometimes these cells were clearly ciliated.

Bronchial glands stained cytoplasmically with variable intensity, some being strongly stained. Perinuclear membranous stain was occasionally noted in glandular epithelial cells. In all tissues scored, lymphocytes were strongly stained.

### *Dysplastic epithelium*

Fifty-six bronchial biopsies with dysplastic epithelium were investigated for expression of the *bcl*-2 protein. None showed the pattern of staining typical of histologically normal epithelium. All had lost the prominent basal layer of stained cells seen in normal epithelium. In two cases dysplastic epithelium was completely negative for *bcl*-2, although lymphocytes in the sections were positive. In 28 cases the intensity of the stain in *bcl*-2-positive cells was weak. In the remaining 26 cases the intensity was similar to or increased compared with *bcl*-2-positive normal epithelium. In all positive dysplasias the proportion and distribution of positive cells was assessed. In six cases only a few positive very weakly stained cells were evident in basal locations. In 37 cases *bcl*-2-positive cells of weak or moderate intensity were found in suprabasal locations and in many cases extended throughout the full thickness of the epithelium. In these cases positive cells were present in varying proportions, from focal positivity to up to approximately two-thirds of the epithelial cells. In 11 cases strong expression was observed in the majority of the epithelial cells and throughout the full thickness. In dysplasias all *bcl*-2-positive cells showed cytoplasmic stain; some showed perinuclear membranous stain. Occasional positive nuclei were observed both in cells in mitosis and in cells not under going mitosis.

Based on the number and distribution of positive cells in the epithelium and their staining intensity, four patterns of *bcl*-2 expression were identified (Table I and Figure 2).

**Table I** Expression of the *bcl*-2 protein in bronchial dysplasia

| | Bcl-2 *staining pattern* *A* | *B* | *C* | *D* |
|---|---|---|---|---|
| Intensity of positive cells | Negative or Weak | Weak | Moderate | Strong |
| Distribution of positive cells | Basal | Up to full thickness | Up to full thickness | Full thickness |
| Proportion of stained cells | <5% | Up to 66% | Up to 66% | >90% |
| Number of cases | 8 | 22 | 15 | 11 |

*Bcl-2 and severity of dysplasia*

Dysplasias of all histological grades showed *bcl*-2 staining patterns A, B and C, while pattern D was only found in severe dysplasia (Table II). Severe dysplasias with the most pleomorphic cells showed the strongest stain. Comparing mild and moderate dysplasias with severe dysplasias, pattern A was more often found in the mild/moderate group ($P = 0.042$), while pattern D was only found in the severe group ($P = 0.004$) (Table II). An increasingly aberrant pattern (from A to D) correlated with an increasing grade of dysplasia (Spearman's rank correlation coefficient of 0.49, $P \leqslant 0.0001$). Thus, staining tended to be more prominent, with a greater proportion of *bcl*-2-positive cells in the upper layers of the epithelium with increasing severity of dysplasia.

In the system of categorisation given in Table I, groups B and C differed only in the staining intensity of *bcl*-2-positive cells. Even if groups B and C were combined in the Spearman rank correlation analysis a trend towards the more aberrant patterns of *bcl*-2 expression was still obtained as severity of dysplasia increased (correlation coefficient of 0.46, $P = 0.0002$).

**Figure 1** Expression of the *bcl*-2 protein in histologically normal bronchial epithelium. Scale bar = 10 μm.

**Figure 2** Expression of the *bcl*-2 protein in dysplastic bronchial epithelium. (**a**) Severe dysplasia showing staining pattern C (scale bar = 10 μm). (**b**) Severe dysplasia showing staining pattern D (scale bar = 20 μm).

*Bcl-2 and p53 expression in dysplastic epithelium*

For many of the samples of dysplastic epithelium examined in this study, expression of p53 had already been investigated (Walker *et al*., 1994). Where sufficient tissue was available, new cases were also examined for p53 expression using the CM1 antibody. There was no correlation between p53 expression and *bcl*-2 expression.

*Bronchial carcinomas*

As described above, the expression of the *bcl*-2 protein was examined in the histologically normal epithelium in bronchial margins from lung carcinoma resections. In the corresponding carcinomas, 20/31 tumours and 16/27 NSCLCs contained *bcl*-2-positive cells (Table III). Eleven out of 31 tumours were completely negative for *bcl*-2, even though lymphoid tissues and, in many sections, adjacent normal epithelium stained strongly. A further 14 tumours had *bcl*-2-negative areas within the tumour. In some *bcl*-2-positive tumours, staining was focal and patchy, although in positive areas stain was evident in all tumour cells. In others *bcl*-2 was expressed more intensely at the periphery of tumour islands. Six tumours showed intense stain throughout the entire tumour. In the remaining positive tumours intensity varied between the tumours and within the tumour sections from weak to strong. In tumours stain was cytoplasmic and in some cases perinuclear membranous. In some tumours occasional cells, either in mitosis or with pleomorphic nuclei, showed intense nuclear staining for *bcl*-2.

Although the number of tumours investigated was small, full clinical information was available for these cases. *Bcl*-2 expression was found in all histological types (Table III). No correlation was found between the degree of differentiation, the UICC stage or TMN score and *bcl*-2 expression.

A scatter diagram for the percentage *bcl*-2-positive tumour cells in the 27 NSCLCs examined in this study is shown in Figure 3. In survival analysis for the NSCLC tumours in this series, the tumour that had 1% tumour cells positive for *bcl*-2 was included in either the negative group or the positive group with no significant difference to the results obtained. No significant survival advantage was shown for patients with tumours which were negative for *bcl*-2 compared with those with *bcl*-2-positive tumours (log-rank test 0–1% *vs* 20–100%, chi-squared 3.43, $P = 0.06$). In contrast, patients in the group whose tumours had 50–100% *bcl*-2-positive tumour cells had a significantly longer survival than those with 0–25% *bcl*-2-positive tumour cells (Figure 4) (log-rank test 0–25% *vs* 50–100%, chi-squared 5.75, $P = 0.02$).

**Table II** *Bcl*-2 expression and severity of displasia

| | *Bcl-2 staining pattern* | | | |
|---|---|---|---|---|
| *Grade of dysplasia* | *A* | *B* | *C* | *D* |
| Mild | 4 | 5 | 2 | 0 |
| Moderate | 2 | 6 | 2 | 0 |
| Severe | 2 | 11 | 11 | 11 |

Mild + moderate *vs* severe: A *vs* B + C + D, $P = 0.042$* (Fisher's exact test); A + B *vs* C + D, $P = 0.004$* (chi-squared test); A + B + C *vs* D, $P = 0.004$* (Fisher's exact test). *Two-tailed probabilities.

Table III Expression of *bcl*-2 in lung carcinomas

| | | Bcl-2 positivity | |
|---|---|---|---|
| *Histology* | *Any tumour cells positive* | *>20% tumour cells positive* | *>50% tumour cells positive* |
| Squamous cell carcinoma ($n = 14$) | 8 | 7 | 5 |
| Adenocarcinomas ($n = 11$) | 6 | 6 | 5 |
| Large-cell carcinomas ($n = 1$) | 1 | 1 | 1 |
| Small-cell carcinomas ($n = 1$) | 1 | 1 | 1 |
| SCLC/squamous carcinomas ($n = 1$) | 1 | 1 | 1 |
| Adenosquamous carcinomas ($n = 1$) | 1 | 1 | 1 |
| Carcinoids ($n = 2$) | 2 | 1 | 0 |
| NSCLC ($n = 27$) | 16 | 15 | 12 |
| Total ($n = 31$) | 20 | 18 | 14 |

Figure 3 Scatter diagram for *bcl*-2 positivity in NSCLC.

Figure 4 Survival of patients with NSCLC in which 50–100% of tumour cells were *bcl*-2 positive compared with those whose tumours contained 0–25% *bcl*-2-positive tumour cells.

*Bcl-2 and p53 expression in bronchial carcinomas*

Immunohistochemical expression of the p53 protein in these tumours had been determined previously (Walker *et al.*, 1994). No correlation was obtained between *bcl*-2 expression and p53 expression.

### Discussion

Although expression of the *bcl*-2 protein has so far been investigated in many haematological malignancies and in a number of solid tumours, few studies have examined the distribution of this protein in premalignant lesions.

The *bcl*-2 protein has been detected by immunohistochemical procedures in a limited number of non-lymphoid tissues under different physiological conditions: in long-lived, post-mitotic cells (neurones), complex organised epithelia (skin and gastric intestinal mucosa) and in glandular epithelium under hormonal control and growth factor control (Hockenbery *et al.*, 1991; McDonnell *et al.*, 1992). In these tissues in which apoptosis accounts for cell turnover, *bcl*-2 is topographically restricted to the long-lived progenitor cells that renew lineages and selected post-mitotic cells that require an extended lifespan (Hockenbery *et al.*, 1991). In previous studies bronchial epithelial mucosa has been reported either not to express *bcl*-2 (Hockenbery *et al.*, 1991) or to show positivity in basal cells, with the more differentiated cells being negative (Lu *et al.*, 1993; Pezzella *et al.*, 1993). We have found that cells with their nuclei in the basal layer of histologically normal bronchial epithelium express *bcl*-2, resulting in a predominantly basal staining pattern. However, although the majority of differentiated cells were negative, in some cases occasional well-differentiated columnar brush border cells also expressed *bcl*-2. The intracellular distribution of the stain in these occasional differentiated bronchial epithelial cells and the compliance with the predicted pattern of *bcl*-2 expression in all other cell types in these sections and control lymphoid tissue suggests that these cells do indeed express *bcl*-2. Furthermore, the monoclonal antibody clone 124 used in this study is reported to have satisfactory specificity and has been used in a number of other studies of both fresh and archival material (Pezzella *et al.*, 1990; Lauwers *et al.*, 1994; Leek *et al.*, 1994; Pilotti *et al.*, 1994; Silvestrini *et al.*, 1994; Ramini and Lu, 1994). These *bcl*-2-positive differentiated cells were seen in the histologically normal epithelium from cancer and non-cancer patients and are therefore unlikely to reflect an early event in the transformation to malignancy. Glandular epithelia such as breast and thyroid (Hockenbery *et al.*, 1991; Pilotti *et al.*, 1994), and in this study bronchial mucosal glands, express *bcl*-2, thus *bcl*-2 positivity in these differentiated bronchial epithelial cells may be related to secretory function.

The *bcl*-2 protein is an integral membrane protein and is usually associated with a cytoplasmic location, being present in mitochondrial membranes, nuclear outer membranes and endoplasmic reticulum (Korsmeyer, 1992; Akao *et al.*, 1994; Lithgow *et al.*, 1994). In this study, occasional cells with *bcl*-2-positive nuclei were noted in bronchial dysplasias and carcinomas. In support of this, in some epithelial cell lines the *bcl*-2 protein has been shown to have occurred transiently in mitotic nuclei (Willingham and Bhalla, 1994; Lu *et al.*, 1994), suggesting that the anti-apoptotic function of *bcl*-2 extends into mitosis.

Expression of the *bcl*-2 protein was altered in all bronchial epithelial dysplasias examined, suggesting that deregulation of *bcl*-2 expression occurs concomitantly with the histological disorganisation that accompanies dysplasia. It is possible that the loss of the prominently stained basal layer seen in histologically normal epithelium and the appearance of *bcl*-2-positive cells in the upper layers of the epithelium in dysplastic lesions results from the change from a pseudostratified columnar to a stratified epithelium in which growth control has become aberrant. It is likely that initial changes in *bcl*-2 expression seen in all dysplasias is secondary to other genetic events which deregulate growth. As *bcl*-2 is associated in normal cells with protection from apoptosis (Korsmeyer, 1992; Kerr *et al*., 1994), *bcl*-2-positive cells in the upper layers of the epithelium may evade stringently controlled normal differentiation and apoptosis and have a growth advantage compared with *bcl*-2-negative cells. While staining patterns A, B and C were found in all histological grades of dysplasia, pattern D was only observed in some of the severe dysplasias. The change or changes resulting in this pattern of *bcl*-2 expression may therefore be a relatively late, but not obligatory event in the progression to invasive neoplasia. This expression pattern may result from a specific genetic lesion in either the *bcl*-2 gene or in a gene which controls *bcl*-2 expression, occurring relatively late in the development of malignancy. t(14; 18) translocations which cause overexpression of *bcl*-2 in some lymphomas (Korsmeyer, 1992) have not been reported in lung cancers, but immunohistochemical overexpression of *bcl*-2 has been reported in follicular lymphomas in the absence of this translocation (Pezzella *et al*., 1990).

Aberrant expression of the *bcl*-2 protein has been reported in other premalignant lesions. In gastric epithelial dysplasias, alterations in the spatial distribution of *bcl*-2-positive cells were observed, with *bcl*-2-positive cells being present in extended regions of the epithelium (Lauwers *et al*., 1994). In premalignant keratinocytic tumours, *bcl*-2 was found to be expressed in 73% of tumours due to Bowen's disease and in 25% of cases of actinic keratosis, in contrast to surrounding *bcl*-2-negative keratinocytes (Nakagawa *et al*., 1994).

In the initial design of this study, a limited number of bronchial tumours were examined to determine whether we obtained a similar distribution of *bcl*-2-positive cells in tumour sections to that reported in the literature for other solid tumours. The *bcl*-2 protein was found to be distributed similarly but present in a higher percentage of the bronchial carcinomas examined in this study than reported by Pezzella *et al*. (1993); however, our tumour numbers were much lower. Because clinical information was available for these cases, analysis of the clinicopathological data was carried out, although the conclusions drawn are limited by the small number of cases examined. *Bcl*-2 was expressed in tumours of all grades of histological differentiation. The only positive correlation obtained was for survival, and this only when survival data for patients with tumours with 0–25% *bcl*-2-positive tumour cells were compared with those for patients with tumours with >50% *bcl*-2-positive cells. Few studies on the prognostic significance of *bcl*-2 in NSCLC are as yet published. In the study by Pezzella *et al*. (1993) a survival advantage for patients with *bcl*-2-positive tumours was found, particularly for patients with squamous cell carcinomas and in those who were over 60 years of age. In this study the *bcl*-2-positive group was compared with the *bcl*-2-negative group (Pezzella *et al*., 1993). It is notable that Silvestrini *et al*. (1994), in their study of the prognostic significance of *bcl*-2 expression in breast carcinomas, compared survival data for patients with more than 30% *bcl*-2-positive cells with those for patients with lower positivity.

The relationship of *bcl*-2 expression to prognosis and survival in non-haematological malignancies is currently poorly defined. In some studies, *bcl*-2 expression is associated with a survival advantage (NSCLC, Pezzella *et al*., 1993; breast, Silvestrini *et al*., 1994) and markers of good prognosis (breast, Leek *et al*., 1994), while in others it has no effect (neuroblastoma, Ramini and Lu, 1994) or is related to poor prognosis (neuroblastoma, Castle *et al*., 1993). While it can be argued that retention of *bcl*-2 expression in tumours may protect against apoptosis and lead to abnormal accumulation of malignant cells (Korsmeyer, 1992; Kerr *et al*., 1994), overexpression of *bcl*-2 has been found to lead to growth inhibition in some cultured cancer cell lines (Pietenpol *et al*., 1994). These observations suggest that further investigation of *bcl*-2 expression in a larger series of NSCLCs than hitherto reported would be justified to assess the clinical utility of this marker.

Both the *bcl*-2 and p53 genes are involved in the genetic control of apoptosis (Haldar *et al*., 1994; Miyashita *et al*., 1994*a*). Recent research has shown that there is a negative response element in the *bcl*-2 gene through which p53 may directly or indirectly transcriptionally down-regulate the *bcl*-2 gene (Miyashita *et al*., 1994*b*). In breast and some thyroid carcinomas and non-Hodgkin's lymphoma, an inverse correlation between the immunohistochemical expression of p53 and *bcl*-2 has been demonstrated (Pezzella *et al*., 1992; Silvestrini *et al*., 1994; Leek *et al*., 1994; Pilotti *et al*., 1994). In contrast, no correlation between p53 and *bcl*-2 expression was observed in this study in either bronchial dysplasias or carcinomas.

In this study, we have shown that abnormal expression of the *bcl*-2 protein is present in bronchial dysplasias of all histological grades, suggesting that changes in the expression of this molecule arise early in the transformation of normal to dysplastic epithelium. Further alterations in its expression may occur late in the progression to malignancy. Before expression of this protein could be used as a biomarker for malignancy, further investigation of the expression of this gene and its biological and prognostic significance in lung cancer is essential.

**Acknowledgements**

We thank Clatterbridge Cancer Research Trust for financial support, Dr N Pendleton for provision of clinical data, and Dr IR Campbell for assistance with statistical analyses.

**References**

AKAO Y, OTSUKI Y, KATAOKA S, ITO Y AND TSUJIMOTO Y. (1994). Multiple subcellular localization of *bcl*-2: detection in nuclear outer membrane, endoplasmic reticulum membrane and mitochondrial membranes. *Cancer Res*., **54**, 2468–2471.

BENNETT WP, COLBY TV, TRAVIS WD, BORKOWSKI A, JONES RT, LANE DP, METCALF RA, SAMET JM, TAKESHIMA Y, GU JR, VÄHÄKANGAS KH, SOINI Y, PAAKKO P, WELSH JA, TRUMP BF AND HARRIS CC. (1993). p53 protein accumulates frequently in early bronchial neoplasia. *Cancer Res*., **53**, 4817–4822.

CANCER RESEARCH CAMPAIGN. (1992). Lung cancer and smoking – UK. Factsheet 11.

CASTLE VP, HEIDELBERGER KP, BROMBERG J, OU X, DOLE M AND NUNEZ G. (1993). Expression of the apoptosis-suppressing protein *bcl*-2, in neuroblastoma is associated with unfavourable histology and N-*myc* amplification. *Am. J. Pathol*., **143**, 1543–1550.

CLEARY ML, SMITH SD AND SKLAR J. (1986). Cloning and structural analysis of cDNAs for *bcl*-2 and a hybrid *bcl*-2/ immunoglobulin transcript resulting from the t(14;18) translocation. *Cell*, **47**, 19–28.

FROST JK, BALL WJ, LEVIN ML, TOCKMAN MS, EROZAN YS, GUPTA PK, EGGLESTON JC, PRESSMAN NJ, DONITHAN MP AND KIMBALL AJ. (1986). Sputum cytopathology: use and potential in monitoring the workplace environment by screening for biological effects of exposure. *J. Occupat. Med*., **28**, 692–703.

GAZDAR A. (1994). The molecular and cellular basis of human lung cancer. *Anticancer Res*., **13**, 261–268.

HALDAR S, NEGRINI M, MONNE M, SABBIONI S AND CROCE CM. (1994). Down-regulation of *bcl*-2 by p53 in breast cancer cells. *Cancer Res*., **54**, 2095–2097.

HIRANO T, FRANZEN B, KATO H, EBIHARA Y AND AUER G. (1994). Genesis of squamous cell lung carcinoma. Sequential changes of proliferation, DNA ploidy and p53 expression. *Am. J. Pathol.*, **144**, 296–302.

HOCKENBERY DM, NUNEZ G, MILLIMAN C, SCHREIBER RD AND KORSMEYER SJ. (1990). *Bcl*-2 is an inner mitochondrial membrane protein that blocks programmed cell death. *Nature*, **348**, 334–336.

HOCKENBERY DM, ZUTTER M, HICKEY W, NAHM M AND KORSMEYER SJ. (1991). *Bcl*-2 protein is topographically restricted in tissues characterized by apoptotic cell death. *Proc. Natl Acad. Sci. USA*, **88**, 6961–6965.

IKEGAKI N, KATSUMATA M, MINNA J AND TSUJIMOTO Y. (1994). Expression of *bcl*-2 in small cell lung carcinoma cells. *Cancer Res.* **54**, 6–8.

JACOBSON MD, BURNE JF, KING MP, MIYASHITA T, REED JC AND RAFF M. (1993). *Bcl*-2 blocks apoptosis in cells lacking mitochondrial DNA. *Nature*, **361**, 365–369.

KERR JF, WINTERFORD CM AND HARMON BV. (1993). Apoptosis. Its significance in cancer and cancer therapy. *Cancer*, **73**, 2013–2026.

KORSMEYER SJ. (1992). *Bcl*-2 initiates a new category of oncogenes: regulators of cell death. *Blood*, **80**, 879–886.

LAUWERS GY, SCOTT GV AND HENDRICKS J. (1994). Immunohistochemical evidence of aberrant *Bcl*-2 protein expression in gastric epithelial dysplasia. *Cancer*, **28**, 2900–2904.

LEE JS, PATHAK S, HOPWOOD V, TOMASOVIC B, MULLINS TD, BAKER FL, SPITZER G AND NEIDHART JA. (1987). Involvement of chromosome 7 in primary lung tumour and nonmalignant normal lung tissue. *Cancer Res.*, **47**, 6349–6352.

LEE JS, LIPPMAN SM, HONG WK, RO JY, KIM SY, LOTAN R AND HITTLEMAN WN. (1992). Determination of biomarkers for intermediate end points in chemoprevention trials. *Cancer Res.*, **52**, 2707s–2710s.

LEEK RD, KALKLAMANIS L, PEZZELLA F, GATTER KC AND HARRIS AL. (1994). *bcl*-2 in normal human breast and carcinoma, association with oestrogen receptor-positive, epidermal growth factor receptor-negative tumours and *in situ* cancer. *Br. J. Cancer*, **69**, 135–139.

LITHGOW T, VANDRIEL R, BERTRAM JF AND STRASSER A. (1994). The protein product of oncogene *Bcl*-2 is a component of the nuclear envelope, the endoplasmic reticulum and the outer mitochondrial membrane. *Cell Growth Different.*, **5**, 411–417.

LOTEM J AND SACHS L. (1993). Regulation by *bcl*-2, *c-myc*, and p53 of susceptibility to induction of apoptosis by heat shock and cancer chemotherapy compounds in differentiation-competent and defective myeloid leukemia cells. *Cell Growth Different.*, **4**, 41–47.

LU Q, POULSOM R, WONG L AND HANBY AM. (1993). *Bcl*-2 expression in adult and embryonic non-haematopoietic tissues. *J. Pathol.*, **169**, 431–437.

LU QL, HANBY AM, HAJIBAGHERI MAN, GSCHMEISSNER SE, LU PJ, TAYLORPAPADIMITRIOU J, KRAJEWSKI S, REED JC AND WRIGHT NA. (1994). *Bcl*-2 protein localizes to the chromosomes of mitotic nuclei and is correlated with the cell cycle in cultured epithelial cell lines. *J. Cell Sci.*, **107**, 363–371.

McDONNELL TJ, TRONCOSO P, BRISBAY SM, LOGOTHETIS C, CHUNG LWK, HSIEH J, TU S AND CAMPBELL ML. (1992). Expression of the protooncogene *bcl*-2 in the prostate and its association with the emergence of androgen-independent prostate cancer. *Cancer Res.*, **52**, 6940–6944.

MIYASHITA T AND REED JC. (1992). *Bcl*-2 gene transfer increases relative resistance of S49.1 and WEH17.2 lymphoid cells to cell death and DNA fragmentation induced by glucocorticoids and multiple chemotherapeutic drugs. *Cancer Res.*, **52**, 5407–5411.

MIYASHITA T, KRAJEWSKI S, KRAJEWSKA M, WANG HG, LIN HK, LIEBERMANN DA, HOFFMAN B AND REED JC. (1994*a*). Tumour suppressor p53 is a regulator of *bcl*-2 and *bax* gene expression *in vitro* and *in vivo*. *Oncogene*, **9**, 1799–1805.

MIYASHITA T, HARIGAI M, HANADA M AND REED JC. (1994*b*). Identification of a p53-dependent negative response element in the *bcl*-2 gene. *Cancer Res.*, **54**, 3131–3135.

NAGAKAWA K, YAMAMURA K, MAEDA S AND ICHIHASHI M. (1994). *Bcl*-2 expression in epidermal keratinocytic diseases. *Cancer*, **74**, 1720–1724.

NUORVA K, SOINI Y, KAMEL D, AUTIO-HARMAINEN H, RISTELI L, RISTELI J, VÄHÄKANGAS K AND PÄKKO P. (1993). Concurrent p53 expression in brochial dysplasias and squamous cell lung carcinomas. *Am. J. Pathol.*, **3**, 725–732.

PENDLETON N, DIXON GR, BURNETT HE, OCCLESTON NL, MYSKOW MW AND GREEN JA. (1993). Expression of proliferating cell nuclear antigen (PCNA) in dysplasia of the bronchial epithelium. *J. Pathol.*, **170**, 169–172.

PEZZELLA F, TSE AGD, CORDELL JL, PULFORD KAF, GATTER KC AND MASON DY. (1990). Expression of the *bcl*-2 oncogene protein is not specific for the 14;18 chromosomal translocation. *Am. J. Pathol.*, **137**, 225–232.

PEZZELLA F, JONES M, RALFKIAER E, ERSBOLL J, GATTER KC AND MASON DY. (1991). Evaluation of *bcl*-2 protein expression and 14;18 translocation as prognostic markers in lymphoma. *Br. J. Cancer*, **65**, 87–89.

PEZZELLA F, MORRISON H, JONES M, GATTER KC, LANE D, HARRIS AL AND MASON DY. (1992). Immunohistochemical detection of p53 and *bcl*-2 proteins in non-Hodgkin's lymphoma. *Histopathology*, **22**, 39–44.

PEZZELLA F, TURLEY H, KUZU I, TUNGEKAR MF, DUNNILL MS, PIERCE CB, HARRIS AH, GATTER KC AND MASON DY. (1993). *Bcl*-2 protein in non-small-cell lung carcinoma. *N. Engl. J. Med.*, **329**, 690–694.

PIETENPOL JA, PAPADOPOULOS N, MARKOWITZ S, WILLSON JKV, KINZLER KW AND VOGELSTEIN B. (1994). Paradoxical inhibition of solid tumour growth by *bcl*-2. *Cancer Res.*, **54**, 3714–3717.

PILOTTI S, COLLINI P, RILKE F, CATTORETTI G, DEL BO R AND PIEROTTI MA. (1994). *Bcl*-2 protein expression in carcinomas originating from the follicular epithelium of the thyroid gland. *J. Pathol.*, **172**, 337–342.

PIRIS MA, PEZZELLA F, MARTINEZ-MONTERO JC, ORRADE JL, VILLUENDAS R, SANCHEZ-BEATO M, CUENA R, CRUZ MA, MARTINEZ B, GARRIDO MC, GATTER K, AIELLO A, DELIA D, GIARDINI R AND RILKE F. (1994). p53 and *bcl*-2 expression in high-grade B-cell lymphomas: correlation with survival time. *Br. J. Cancer*, **69**, 337–341.

RAMINI P AND LU Q. (1994). Expression of *bcl*-2 gene product in neuroblastoma. *J. Pathol.*, **172**, 273–278.

ROTH JA. (1992). New approaches to treating early lung cancer. *Cancer Res.*, **52**, 2652s–2657s.

SEGAL NH, COHEN RJ, HAFFEJEE Z AND SAVAGE N. (1994). *Bcl*-2 protooncogene expression in prostate cancer and its relationship to the prostatic neuroendocrine cell. *Archives Pathol. Lab. Med.*, **118**, 616–618.

SENTMEN CL, SHUTTER JR, HOCKENBERY D, KANAGAWA O AND KORSMEYER SJ. (1991). *Bcl*-2 inhibits multiple forms of apoptosis but not negative selection in thymocytes. *Cell*, **29**, 879–888.

SILVESTRINI R, VENERONI S, DAIDONE MG, BENINI E, BORACCHI P, MEZZETTI M, DI FRONZO G, RILKE F AND VERONESI U. (1994). The *bcl*-2: a prognostic indicator strongly related to p53 protein in lymph node-negative breast cancer patients. *J. Natl Cancer Inst.*, **86**, 499–504.

SOUHAMI R. (1992). Lung cancer. *Br. Med. J.*, **304**, 1298–1301.

SOZZI G, MIOZZO M, TAGLIABUE E, CALDERONE C, LOMBARDI L, PILOTTI S, PASTORINO U, PIEROTTI MA AND DELLA PORTA G. (1991). Cytogenetic abnormalities and overexpression of receptors for growth factors in normal bronchial epithelium and tumor samples of lung cancer patients. *Cancer Res.*, **51**, 400–404.

SUNDARESAN V, GANLY P, HASLETON P, RUDD R, SINHA G, BLEEHEN NM AND RABBITTS P. (1992). p53 and chromosome 3 abnormalities, characteristic of lung tumours, are detectable in preinvasive lesions of the bronchus. *Oncogene*, **7**, 1989–1997.

TSUJIMOTO Y. (1989). Stress-resistance conferred by high level of *bcl*-2α protein in human B lymphoblastoid cell. *Oncogene*, **4**, 1331–1336.

TSUJIMOTO Y AND CROCE CM. (1986). Analysis of the structure, transcripts and protein products of *bcl*-2 the gene involved in human follicular lymphoma. *Proc. Natl Acad. Sci. USA*, **83**, 5214–5218.

TSUJIMOTO Y, COSSMAN J, JAFFE E AND CROCE CM. (1985). Involvement of the *bcl*-2 oncogene in human follicular lymphoma. *Science*, **228**, 1440–1443.

TOCKMAN MS, GUPTA PK, PRESSMAN NJ AND MULSHINE JL. (1992). Considerations in bringing a cancer biomarker to clinical application. *Cancer Res.*, **52**, 2711s–2718s.

WALKER C, ROBERTSON LJ, MYSKOW MW, PENDLETON N AND DIXON GR. (1994). p53 expression in normal and dysplastic bronchial epithelium and in lung carcinomas. *Br. J. Cancer*, **70**, 297–303.

WILLINGHAM MC AND BHALLA K. (1994). Transient mitotic phase localization of *bcl*-2 oncoprotein in human carcinoma cells and its possible role in prevention of apoptosis. *J. Histochem. Cytochem.*, **42**, 441–450.